# Switching at Low HIV-1 RNA into Fixed Dose Combinations: TDF/FTC/RPV is non-inferior to TDF/FTC/EFV in first-line suppressed patients living with HIV

**DOI:** 10.4102/sajhivmed.v20i1.949

**Published:** 2019-07-23

**Authors:** Paula Munderi, Edwin Were, Anchalee Avihingsanon, Pascale A.M. Mbida, Lerato Mohapi, Samba B. Moussa, Marjolein Jansen, Ceyhun Bicer, Perry Mohammed, Yvon van Delft

**Affiliations:** 1 MRC/UVRI Uganda Research Unit on AIDS, Entebbe, Uganda; 2 Partners in Prevention, Eldoret, Kenya; 3 HIV Netherlands Australia Thailand Research Collaboration (HIV-NAT), Thailand; 4 Faculty of Medicine, Chulalongkorn University, Bangkok, Thailand; 5 Cabinet Medical IDOC, Douala, Cameroon; 6 Perinatal HIV Research Unit, University of the Witwatersrand, Johannesburg, South Africa; 7 District Centre de Gaspard Kamara, Dakar, Senegal; 8 Janssen-Cilag BV, Breda, the Netherlands; 9 BICER Consulting & Research, Antwerp, Belgium; 10 Janssen Ltd, High Wycombe, United Kingdom

**Keywords:** LMIC, Single-Tablet-Regimen, Virologically suppressed adults, Treatment-emergent Resistance, SALIF

## Abstract

**Background:**

In low- and middle-income countries (LMICs), a substantial unmet need for affordable single-tablet regimen (STR) options remains. Rilpivirine (RPV, TMC278) is formulated in a low-cost STR with tenofovir disoproxil fumarate (TDF) and emtricitabine (FTC).

**Objectives:**

Switching at Low HIV-1 RNA into Fixed Dose Combinations (SALIF) compared RPV with efavirenz (EFV), both as STRs with TDF and FTC, in maintaining virologic suppression.

**Methods:**

SALIF was a phase 3b, randomised, open-label, non-inferiority study in virologically suppressed adults (HIV-1 RNA < 50 copies/mL) on non-nucleoside reverse transcriptase inhibitor (NNRTI)-based first-line antiretroviral therapy (ART) in Cameroon, Kenya, Senegal, South Africa, Uganda and Thailand. Patients (*N* = 426), stratified by NNRTI use, were randomised 1:1 to receive TDF/FTC/RPV (300/200/25 mg qd) or TDF/FTC/EFV (300/200/600 mg qd). Primary endpoint was proportion of patients with virologic suppression (HIV-1 RNA < 400 copies/mL) at week 48 (intent-to-treat, modified Food and Drug Administration Snapshot, 10% non-inferiority margin).

**Results:**

Patients received TDF/FTC/RPV (*n* = 213) or TDF/FTC/EFV (*n* = 211). At week 48, virologic suppression was maintained in 200/213 (93.9%) patients in the RPV arm and 203/211 (96.2%) in the EFV arm (difference –2.3%; 95% confidence interval: −6.4, +1.8), demonstrating non-inferiority of TDF/FTC/RPV. One patient in each arm experienced virologic failure without treatment-emergent resistance. Twenty-seven patients discontinued prematurely (8.0% RPV vs. 4.7% EFV), the most frequent reasons being adverse events (3.3% vs. 0.5%, respectively), site closure (1.9% vs. 0.5%), loss to follow-up (0.9% vs. 1.4%) and consent withdrawal (0.9% vs. 1.4%).

**Conclusion:**

In adults with suppressed viral load on first-line NNRTI-based ART in LMICs, switching to an STR of TDF/FTC/RPV was non-inferior to TDF/FTC/EFV in maintaining high rates of viral suppression with a comparable tolerability profile.

## Introduction

Current HIV treatment guidelines^1,2,3^ recommend that antiretroviral therapy (ART), administered as a single-tablet regimen (STR), can be initiated in all patients living with HIV, regardless of clinical stage and CD4+ cell count. In low- and middle-income countries (LMICs), there is a substantial unmet need for affordable STR options. The WHO policy brief from July 2018^4^ stated that dolutegravir (DTG)-based regimens may be recommended as a preferred first-line regimen for people living with HIV initiating ART, and the alternative first-line treatment regimen is efavirenz (EFV)-based. These first-line recommended treatments may result in some patients experiencing neuropsychiatric events or other tolerability issues,^5,6,7^ while the use of nevirapine (NVP) is associated with the risk of hepatotoxicity and skin reactions.^8^ Given that DTG is not recommended for use in non-pregnant women who are trying to conceive or during the first trimester of pregnancy because of concerns about a possible increased risk of neural tube defects,^9^ a viable alternative to DTG is needed. There is, thus, an unmet need for additional efficacious, well-tolerated and more affordable alternative ART regimens, particularly in LMICs.^10^

Rilpivirine (RPV; TMC278) is a second-generation non-nucleoside reverse transcriptase inhibitor (NNRTI) with a good safety profile^11^ and convenient once-daily dosing, with a reduced special effort access price of US$40 per patient per year in sub-Saharan Africa and least developed countries,^12^ making it a good candidate component for an affordable STR in LMICs.^13,14,15,16,17^ RPV, in combination, is indicated in treatment-naïve patients 12 years of age and older with a viral load of HIV-1 RNA ≤ 100,000 copies/mL.^18^ Tenofovir disoproxil fumarate (TDF)/lamivudine (3TC) or emtricitabine (FTC) with RPV is listed as a ‘preferred’ first-line regimen in European guidelines,^3^ has recently been adopted as a preferred first-line regimen in South African guidelines^19^ and is a recommended ‘alternative’ regimen in the United States and Thailand.^2,20^

Approval of RPV was based on findings from two double-blind, placebo-controlled, phase 3 trials, ECHO and THRIVE, comparing RPV with EFV, most commonly in combination with TDF and FTC, in treatment-naive patients.^6,7,21^ In a 48-week pooled analysis of these trials, RPV was non-inferior to EFV both in patients with viral loads ≤ 100 000 copies/mL (90% vs. 84%; 95% confidence interval [CI]: +1.6, +11.5) and with viral loads > 100 000 to ≤ 500 000 copies/mL (80% vs. 83%; 95% CI: –9.8, +3.7),^21^ but non-inferiority was not achieved for patients with viral loads > 500 000 copies/mL (70% vs. 76%; 95% CI: –20.4, +8.30). In addition to these pivotal trials using the individual agents, the STR of TDF/FTC/RPV has been evaluated in both treatment-naive patients and virologically suppressed patients, including in at least one LMIC setting. In these studies, TDF/FTC/RPV was found to be non-inferior to several different ART regimens, including protease inhibitor (PI)- and NNRTI-based combinations.^22,23,24,25^ Hence, in Europe and the United States, TDF/FTC/RPV is indicated for use in treatment-naive patients with HIV-1 RNA ≤ 100 000 copies/mL and for patients with suppressed viral load for ≥ 6 months prior to switching therapy and without known resistance-associated mutations to NNRTIs, TDF or FTC.^26,27^

Preclinical studies of RPV showed no evidence of teratogenicity RPV and therefore may be an option in populations with large proportions of HIV-infected women of childbearing potential who have access to regular viral load monitoring.^28^ Pharmacokinetic studies of RPV in pregnancy reveal that most women achieve effective plasma concentrations of RPV.^29,30,31^ The Antiretroviral Pregnancy Registry showed no increased risk in birth defects after first trimester RPV exposures as of January 2018.^32^ Furthermore, according to both the US Department of Health and Human Services Recommendations for Use of Antiretroviral Drugs in Pregnant HIV-1-Infected Women and the 2017 European AIDS Clinical Society guidelines, there are sufficient data from use in pregnancy to recommend RPV as an alternative agent in ART-naive pregnant women with viral loads ≤ 100 000 copies/mL and CD4+ counts > 200 cells/mm^3^.^3,33^

This study was, therefore, designed to examine the utility of switching to the STR of TDF/FTC/RPV in LMIC patients with suppressed viral loads, who were on an NNRTI-based first-line ART.

## Methods

Switching at Low HIV-1 RNA into Fixed Dose Combinations (SALIF) was a 48-week, multicentre, phase 3b, randomised, open-label study designed to demonstrate non-inferiority of RPV to EFV (both coformulated with TDF and FTC) in maintaining HIV-1 RNA suppression (defined as HIV-1 RNA < 400 copies/mL) among adult patients in LMICs on first-line NNRTI-based ART (with EFV or NVP) with HIV-1 RNA < 50 copies/mL. The study was conducted at 23 sites in Cameroon, Kenya, Senegal, South Africa, Uganda and Thailand from 23 August 2013 to 27 October 2015.

Ethics committee approval was obtained at all participating centres in accordance with the principles of the 2008 Declaration of Helsinki. Central randomisation was based on a computer-generated schedule prepared before the study by the sponsor. Randomisation was balanced by using randomly permuted blocks and stratified by baseline NNRTI. A pre-specified interim analysis was performed once all patients had reached week 24 or discontinued earlier, and was reviewed by an independent data monitoring committee. Data from this study have been presented previously.^34,35^

All patients remained on study until the last patient reached the week 48 visit. Patients were then switched to an investigator-selected treatment according to local prescribing practice. In countries where a RPV-based regimen was not yet available, patients with suppressed HIV-1 RNA levels receiving TDF/FTC/RPV could continue in post-trial access programmes until RPV was publicly available in the country.

### Study patients

The study included adults (≥ 18 or 21 years of age, depending on national legislation of patient’s country) with documented HIV-1 infection, who had been receiving first-line NNRTI-based ART (defined as two nucleoside reverse transcriptase inhibitors [NRTIs] with either EFV or NVP) for at least 1 year, and the same ART for at least 8 weeks, before screening. Previous changes in NRTI background regimen were allowed, but patients who had previously switched from EFV to NVP for toxicity reasons were not eligible. At screening, eligible patients had to have suppressed viral loads, commonly accepted to be a plasma HIV-1 RNA < 50 copies/mL,^36^ a CD4+ cell count of more than 200 cells/mm^3^, and a preference to change their current ART for reasons of simplification and/or NRTI toxicity. Patients also needed to have access to at least one meal a day and have a normal electrocardiogram (ECG) to be eligible. Patients co-infected with *Mycobacterium tuberculosis*, who were likely to require rifampicin-based treatment during the study, were excluded. Written informed consent was obtained from each patient prior to the screening procedures.

### Treatment

At baseline, patients were randomly assigned (1:1) to receive an STR of either TDF (300 mg)/FTC (200 mg)/RPV (25 mg) or TDF (300 mg)/FTC (200 mg)/EFV (600 mg). Both products were supplied by the sponsor and given in accordance with the product labels at the recommended dose of one tablet per day. Patients randomised to TDF/FTC/RPV were advised to take the medication with food, whereas patients randomised to TDF/FTC/EFV were advised to take it on an empty stomach at bedtime. To assess adherence, patients were asked to bring the study drug containers, whether empty or not, to each study visit.

### Assessments

Blood samples were collected at screening, baseline, weeks 4, 12, 24, 36 and 48, and every 24 weeks up to study end or until discontinuation and then at post-treatment follow-up. HIV-1 RNA was measured at a central laboratory, using the Abbott RealTi*m*e HIV-1 RNA assay with a lower limit of quantification of 40 copies/mL. Patients with a plasma HIV-1 RNA level ≥ 50 copies/mL were counselled on treatment adherence, and had blood samples collected for re-testing at the central laboratory at up to 8-week intervals until the plasma HIV-1 RNA was < 50 copies/mL or the plasma HIV-1 RNA level was confirmed by two consecutive tests to be ≥ 400 copies/mL. Patients with a confirmed plasma HIV-1 RNA level ≥ 400 copies/mL measured at the central laboratory were classified as virologic failures. The confirmatory viral load sample was tested for genotypic drug resistance at the central laboratory.

CD4+ cell counts were determined at a central laboratory at screening, baseline, every 24 weeks up to study end or until discontinuation and then at post-treatment follow-up.

### Safety

Safety monitoring (adverse events [AEs], including HIV-related events, clinical laboratory analyses, vital signs and physical examination) was performed throughout the treatment phase until study end. Electrocardiograms were recorded at screening, weeks 24 and 48, or at treatment discontinuation if earlier. The following AE classes of interest were investigated based on previous data from the RPV pivotal studies: rashes, neuropsychiatric events, potential QT prolongation-related events, hepatic events and endocrinological events. In addition, hyperglycaemia and new onset diabetes were analysed based on reported AEs during the study.

### Statistical analysis and endpoints

The primary objective was to demonstrate non-inferiority of a TDF/FTC/RPV STR versus TDF/FTC/EFV STR in the percentage of patients with plasma HIV-1 RNA levels < 400 copies/mL after 48 weeks (non-inferiority margin of 10%) using a modified Food and Drug Administration (FDA) Snapshot method.^31^ Patients were classified as virologic responders if their HIV-1 RNA was < 400 copies/mL within the time window of the week 48 visit (between week 42 and week 58), or if a single HIV-1 RNA value ≥ 400 copies/mL within the time window was not confirmed by a second measurement – the definition of virologic suppression selected was < 400 copies/mL, to reflect the real-life practice in LMICs where a viral load of < 1000 copies/mL should be taken as evidence as suppression.^37^ Patients with no HIV-1 RNA measurement within the time window of the week 48 visit were considered non-responders.

Secondary endpoints were non-inferiority in the percentage of patients with plasma HIV-1 RNA levels < 50 copies/mL after 48 weeks (modified FDA Snapshot method), rates of virologic failure during the 48 weeks of treatment with HIV-1 RNA levels ≥ 400 or ≥ 50 copies/mL (non-virologic failure-censored analysis excluding patients who discontinued the study with HIV-1 RNA < 400 or < 50 copies/mL), change in CD4+ cell count, loss of treatment options, as defined by treatment-emergent drug resistance, and adherence to study treatment based on tablet count at each study visit up to week 48.

Assuming response rates of 90% at 48 weeks for both treatment arms, 192 patients were required per arm to establish non-inferiority of TDF/FTC/RPV versus TDF/FTC/EFV, with a maximum allowable difference of 10%, a one-sided significance level of 2.5%, and 90% power. To account for a maximum of up to 10% major protocol deviations that would result in exclusion of patients from the per protocol (PP) analysis, 213 patients were planned to be recruited in each treatment arm, resulting in 426 randomised patients in total.

The primary efficacy analysis was conducted on the intent-to-treat (ITT) population (all randomised patients who had taken at least one dose of study drug, regardless of their compliance with the protocol). This analysis was repeated for the PP population (a subset of the ITT population that excluded patients with major protocol deviations). As pre-specified in the statistical analysis plan (SAP), treatment arms were compared using the Cochran–Mantel–Haenszel method, adjusted for the stratification variable (use of EFV vs. NVP at the screening visit). TDF/FTC/RPV was considered non-inferior to TDF/FTC/EFV if the lower limit of the 95% CI of the difference in efficacy was ≥ 10%. Analysis of the percentages of patients with HIV-1 RNA levels < 50 copies/mL, a secondary efficacy outcome, used the same statistical methods as the primary analysis.

Subgroup analyses of the virologic response were performed in the ITT population for the following pre-defined groups: NNRTI taken at screening (as stratified), baseline CD4+ count category, sex, country and treatment adherence. The ITT population was used for all safety analyses; as pre-specified in the SAP, there was no formal statistical testing of safety parameters in the study.

## Ethical consideration

Ethics committee approval was obtained at all participating centres in accordance with the principles of the 2008 Declaration of Helsinki.

## Results

### Study patients

**FIGURE 1:**
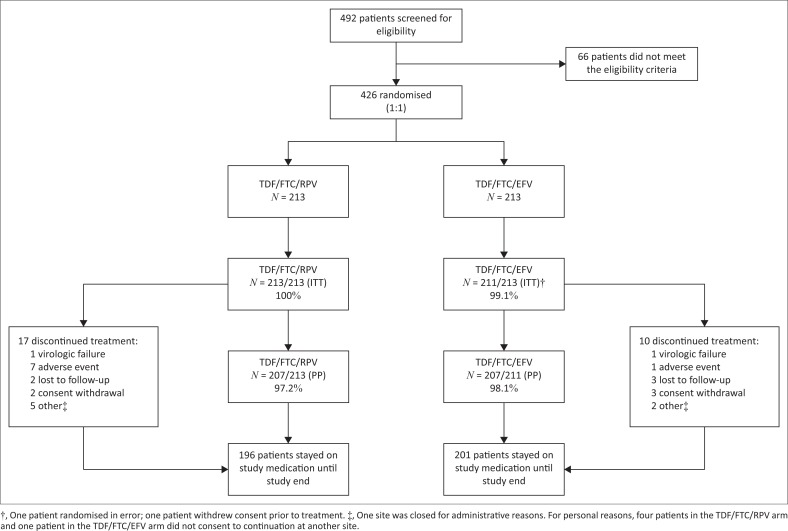
Patient disposition.

Patients were recruited between 23 August 2013 and 14 August 2014. Treatment duration was between 48 and 108 weeks. Of 492 patients screened, 66 were excluded and 426 were randomised (213 in each arm); two patients in the TDF/FTC/EFV arm did not start randomised therapy (one was randomised in error and one withdrew consent). The ITT population comprised 424 patients (Figure 1).

Overall, demographic and baseline disease characteristics were well balanced between the two treatment arms (Table 1). Most patients (309/424 [72.9%]) were black people and of African origin (315/424 [74.3%]), with 271/424 (63.9%) being women, mostly of childbearing age.

**TABLE 1:** Baseline and disease characteristics.

Characteristic	TDF/FTC/RPV (*n* = 213)				TDF/FTC/EFV (*n* = 211)				All patients (*n* = 424)			
	*n*	*N*	%	s.d.	*n*	*N*	%	s.d.	*n*	*N*	%	s.d.
Female	137	-	64.3	-	134	-	63.5	-	271	-	63.9	-
Women of childbearing potential	98	137	71.5	-	97	134	72.4	-	195	271	72.0	-
Mean age, years	40.6	-	-	8.0	40.6	-	-	8.7	40.6	-	-	8.3
**Race**												
Black people	157	-	73.7	-	152	-	72.0	-	309	-	72.9	-
Asian	51	-	23.9	-	58	-	27.5	-	109	-	25.7	-
Other	5	-	2.3	-	1	-	0.5	-	6	-	1.4	-
**Nationality**												
Cameroon	16	-	7.5	-	13	-	6.2	-	29	-	6.8	-
Kenya	36	-	16.9	-	37	-	17.5	-	73	-	17.2	-
Senegal	17	-	8.0	-	8	-	3.8	-	25	-	5.9	-
South Africa	33	-	15.5	-	30	-	14.2	-	63	-	14.9	-
Thailand	51	-	23.9	-	58	-	27.5	-	109	-	25.7	-
Uganda	60	-	28.2	-	65	-	30.8	-	125	-	29.5	-
**Mean BMI (k g/m^2^)**	24.25	-	-	4.8	24.11	-	-	5.0	24.18	-	-	4.9
**Mode of HIV infection**												
Heterosexual contact	188	-	88.3	-	188	-	89.1	-	376	-	88.7	-
Men having sex with men	13	-	6.1	-	16	-	7.6	-	29	-	6.8	-
Other	12	-	5.6	-	7	-	3.3	-	19	-	4.5	-
Mean time since diagnosis, years	7.6	-	-	4.6	8.2	-	-	4.8	7.9	-	-	4.7
Mean time since first ART, years	5.8	-	-	3.3	6.0	-	-	3.3	5.9	-	-	3.3
Mean CD4+ cell count, cells/mm³	545.3	-	-	228.2	549.3	-	-	207.7	547.3	-	-	218.0
**Hepatitis reactive**												
Hepatitis B surface antigen	12	-	5.6	-	10	-	4.7	-	22	-	5.2	-
Hepatitis C antibody	3	-	1.4	-	5	-	2.4	-	8	-	1.9	-
**NNRTI at screening**												
EFV	115	-	54.0	-	116	-	55.0	-	231	-	54.5	-
NVP	98	-	46.0	-	95	-	45.0	-	193	-	45.5	-
**NRTI at screening**												
3TC + ZDV	89	-	41.8	-	89	-	42.2	-	178	-	42.0	-
3TC + TDF	117	-	54.9	-	112	-	53.1	-	229	-	54.0	-
Other	7	-	3.3	-	10	-	4.7	-	17	-	4.0	-

3TC, lamivudine; ART, antiretroviral therapy; BMI, body mass index; EFV, efavirenz; FTC, emtricitabine; NNRTI, non-nucleoside reverse transcriptase inhibitor; NRTI, nucleoside reverse transcriptase inhibitor; NVP, nevirapine; RPV, rilpivirine; s.d., standard deviation; TDF, tenofovir disoproxil fumarate; ZDV, zidovudine.

All patients were taking EFV- or NVP-based regimen at screening. Most patients (415/424 [97.9%]) were taking a non-STR before being enrolled in the study. After randomisation, in the TDF/FTC/EFV arm, 116/211 (55.0%) patients remained on EFV and 95/211 (45.0%) patients changed their NVP for EFV. In the TDF/FTC/RPV arm, 115/213 (54.0%) patients were taking an EFV-based regimen and 98/213 (46.0%) patients were taking an NVP-based regimen at screening. In the TDF/FTC/RPV arm, all (213 [100.0%]) patients changed their NNRTI to RPV at randomisation. In addition, all but two patients (99.1%) had a switch in both their NNRTI and NRTI, while in the TDF/FTC/EFV arm, only 45.5% had to switch both their NNRTI and NRTI. In total, 397 (93.6%) of 424 patients stayed on study medication until study end. Of the patients who discontinued prematurely, 17/213 (8.0%) had received RPV and 10/211 (4.7%) had received EFV. High adherence rates (more than 95% adherence based on tablet count) were documented in 95.8% (204/213) switched to TDF/FTC/RPV and in 97.6% (206/211) switched to TDF/FTC/EFV.

### Efficacy

**FIGURE 2:**
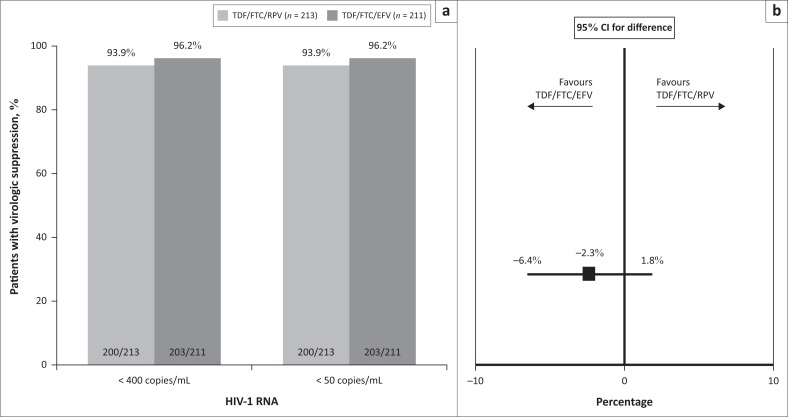
(a) Comparison of TDF/FTC/RPV and TDF/FTC/EFV showing a) % of patients with plasma HIV-1 RNA < 400 copies/mL (primary endpoint) and < 50 copies/mL at week 48 (ITT, modified FDA Snapshot analysis) and (b) difference in the primary endpoint between the two arms demonstrating non-inferiority.

**FIGURE 3:**
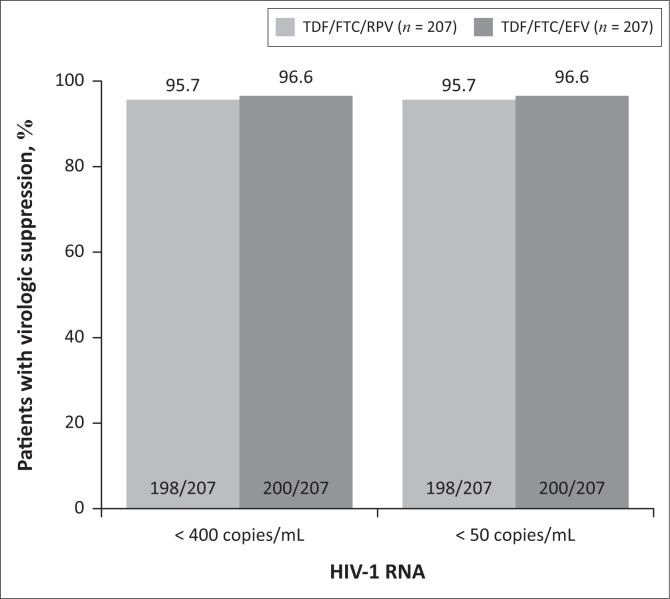
Plasma HIV-1 RNA < 400 and < 50 copies/mL at week 48 (PP population, modified FDA Snapshot).

The primary endpoint of HIV-1 RNA < 400 copies/mL at week 48 (ITT, modified FDA Snapshot analysis) was reached by 200/213 (93.9%) patients in the TDF/FTC/RPV arm and 203/211 (96.2%) patients in the TDF/FTC/EFV arm with a difference of –2.3% (95% CI: –6.44, +1.84), demonstrating non-inferiority of TDF/FTC/RPV (*p* = 0.0003) (Figure 2). In the PP population, virologic suppression was achieved by 198/207 (95.7%) patients in the TDF/FTC/RPV arm and 200/207 (96.6%) patients in the TDF/FTC/EFV arm (difference 0.9%, 95% CI: –4.66, +2.72) (Figure 3). The results for the secondary endpoint of HIV-1 RNA < 50 copies/mL at week 48 were identical to those for the primary endpoint for both ITT and PP populations (Figures 2 and 3).

In the ITT population, 13/213 patients (6.1%) in the TDF/FTC/RPV arm were non-responders at week 48. Among the non-responders, five patients discontinued because of AEs, seven for other reasons and one patient experienced virologic failure. In the TDF/FTC/EFV arm, 8/211 patients (3.8%) were non-responders at week 48: one patient discontinued because of AEs, six for other reasons and one patient experienced virologic failure. Four patients in the TDF/FTC/RPV arm and one patient in the TDF/FTC/EFV arm who discontinued for ‘other reasons’ did so because they did not re-consent to continue the study at another site after their initial site closed because of administrative reasons. In the PP population, 9/207 patients (4.3%) and 7/207 patients (3.4%) were non-responders in the TDF/FTC/RPV and TDF/FTC/EFV group, respectively.

The mean (s.d.) increase in CD4+ cell count from baseline at week 48 was 26.2 (125.14) cells/mm^3^ in the TDF/FTC/RPV group and 6.1 (140.06) cells/mm^3^ in the TDF/FTC/EFV group.

No resistance-associated mutations of the pre-defined list (IAS-USA NRTI, IAS-USA NNRTI, extended NNRTI or RPV resistance-associated mutations or primary IAS-USA PI mutations) were detected in the samples from two patients who had experienced virologic failure, thus there was no loss of treatment options observed in this study.

### Subgroup analyses

For patients receiving EFV-based regimens at screening, a virologic response of HIV-1 RNA < 400 copies/mL at week 48 was achieved by 114/116 (98.3%) patients who remained on EFV and 107/115 (93.0%) of those who switched to RPV (difference –5.2%; 95% CI: –10.45, –0.01). For patients receiving NVP-based regimens at screening, the corresponding virologic response rate was 93.7% (89/95) in the TDF/FTC/EFV arm and 94.9% (93/98) in the TDF/FTC/RPV arm (difference 1.2%; 95% CI: –5.34, +7.76). The study was not powered to detect non-inferiority between sub groups of the NNRTI at screening. No major differences were observed when stratifying virologic response by sex, baseline CD4+ cell count, adherence or country (Table 2).

**TABLE 2:** Virologic response (HIV-1 RNA <400 copies/mL) by subgroups at week 48 (ITT, modified FDA Snapshot).

Variable	TDF/FTC/RPV			TDF/FTC/EFV			Difference (%)	95% CI
	*n*	*N*	%	*n*	*N*	%		
**NNRTI at screening**								
EFV	107	115	93.0	114	116	98.3	−5.2	−10.45, −0.01
NVP	93	98	94.9	89	95	93.7	1.2	−5.34, +7.76
**Baseline CD4+**								
< 200 cells/mm^3^	1	1	100	3	3	100	0	0.00, 0.00
200–349 cells/mm^3^	39	41	95.1	25	26	96.2	−1.0	−10.94, +8.87
≥ 350 cells/mm^3^	160	171	93.6	175	182	96.2	−2.6	−7.20, +2.03
**Sex**								
Female	131	137	95.6	129	134	96.3	−0.6	−5.34, +4.05
Male	69	76	90.8	74	77	96.1	−5.3	−13.12, +2.49
**Country**								
All African countries	153	162	94.4	147	153	96.1	−1.6	−6.31, +3.05
Cameroon	16	16	100	13	13	100	0	0.00, 0.00
Kenya	35	36	97.2	35	37	94.6	2.6	−6.42, +11.68
Senegal†	11	17	64.7	7	8	87.5	−22.8	−55.06, +9.47
South Africa	32	33	97.0	28	30	93.3	3.6	−7.04, +14.31
Uganda	59	60	98.3	64	65	98.5	−0.1	−4.54, +4.28
Thailand	47	51	92.2	56	58	96.6	−4.4	−13.14, +4.35
**Adherence**								
> 95%	192	204	94.1	199	206	96.6	−2.5	−6.55, +1.58
≤ 95%	8	9	88.9	4	5	80.0	8.9	−31.74, +49.52

CI, confidence interval; EFV, efavirenz; FDA, Food and Drug Administration; FTC, emtricitabine; ITT, intent-to-treat; NNRTI, non-nucleoside reverse transcriptase inhibitor; NVP, nevirapine; RPV, rilpivirine; TDF, tenofovir disoproxil fumarate.

† , One of the clinical sites in Senegal was closed before the end of the trial. This led to some patients not being followed up for the full 48 weeks and thus a lower response rate in the ITT Snapshot analysis.

### Safety

Safety data were collected for all patients up until study end. There were no relevant differences in the incidence of AEs between the two arms, except for Division of AIDS (DAIDS) grade 3 or 4 AEs which were more commonly reported in patients receiving TDF/FTC/EFV compared with TDF/FTC/RPV (56 [26.5%] vs. 40 [18.8%], respectively). Regardless of NNRTI at screening, the incidence of treatment-emergent DAIDS grade 3 or 4 AEs was lower in the TDF/FTC/RPV arm compared with TDF/FTC/EFV. For patients who switched from EFV at baseline, the incidence was 15.7% (18/115) in the TDF/FTC/RPV arm versus 24.1% (28/116) in the TDF/FTC/EFV arm; for those patients who switched from NVP at baseline, the incidence was 22.4% (22/98) versus 29.5% (28/95), respectively.

The most frequently reported AEs at least possibly related to the study drugs were vertigo (5 [2.3%] in the TDF/FTC/RPV arm vs. 11 [5.2%] in the TDF/FTC/EFV arm), headache (10 [4.7%] vs. 6 [2.8%]), dizziness (2 [0.9%] vs. 8 [3.8%]), increased appetite (8 [3.8%] vs. 1 [0.5%]) and nightmares (0 vs. 8 [3.8%]) (Table 3).

**TABLE 3:** Clinical adverse events and laboratory abnormalities.

Adverse event	TDF/FTC/RPV (*n* = 213)		TDF/FTC/EFV (*n* = 211)	
	*n*	%	*n*	%
Any adverse event	178	83.6	174	82.5
Drug-related adverse events (all grades)	65	30.5	53	25.1
**Drug-related adverse events (all grades) in ≥ 1% of patients in either treatment arm**				
Headache	10	4.7	6	2.8
Dizziness	2	0.9	8	3.8
Vertigo	5	2.3	11	5.2
Insomnia	5	2.3	1	0.5
Nightmares	-	-	8	3.8
Peripheral neuropathy	4	1.9	2	0.9
Generalised pruritus	2	0.9	3	1.4
Increased amylase	5	2.3	-	-
Nausea	5	2.3	2	0.9
Diarrhoea	1	0.5	4	1.9
Increased appetite	8	3.8	1	0.5
Renal and urinary disorders	1	0.5	5	2.4
Grade 3–4 adverse events	40	18.8	56	26.5
Drug-related grade 3–4 adverse events	13	6.1	4	1.9
**Drug-related grade 3–4 adverse events in ≥ 1% of patients in either treatment arm**				
Amylase increased	5	2.3	-	-
Alanine aminotransferase increased	3	1.4	-	-
Serious adverse events	16	7.5	11	5.2
Drug-related serious adverse events	3	1.4	1	0.5
Deaths	1	0.5†	-	-
Discontinuations because of adverse events	7	3.3‡	1	0.5§
**Select grade 3–4 laboratory abnormalities**				
Amylase	6	2.8	11	5.3
Alanine aminotransferase	4	1.9	2	0.9
Aspartate aminotransferase	1	0.5	1	0.5
Total cholesterol	-	-	4	1.9
LDL cholesterol	2	0.9	11	5.2
Triglycerides	-	-	1	0.5
Hyperglycaemia	3	1.4	-	-

ALT, alanine aminotransferase; EFV, efavirenz; FTC, emtricitabine; LDL, low-density lipoprotein; RPV, rilpivirine; TDF, tenofovir disoproxil fumarate.

† , Myocardial infarction, unrelated to study medication;

‡ , Elevated ALT grade 3 (*n* = 2), QT prolongation grade 3 (*n* = 2), increased creatinine (*n* = 1), tachycardia (*n* = 1), tuberculosis (*n* = 1);

§ , Lipoatrophy.

More patients in the TDF/FTC/EFV arm than in the TDF/FTC/RPV arm experienced grade 3 or higher lipid abnormalities (total cholesterol: 0 in the TDF/FTC/RPV arm vs. 4 [1.9%], low-density lipoprotein [LDL] cholesterol: 2 [0.9%] vs. 11 [5.2%], triglycerides: 0 vs. 1 [0.5%]).

Adverse events leading to discontinuation were reported in seven (3.3%) patients switched to TDF/FTC/RPV and in one (0.5%) patient receiving TDF/FTC/EFV. All AEs leading to permanent discontinuation were observed in at most one patient in any treatment arm, except for alanine aminotransferase increases and ECG QT prolongation, which both occurred in two (0.9%) patients in the TDF/FTC/RPV arm.

In the TDF/FTC/EFV group, a lower rate of treatment-emergent neuropsychiatric events of interest was seen in the subgroup of patients who were receiving EFV at screening (20/116 patients, 17.2%) compared with the rate in patients receiving NVP at screening (39/95 patients, 41.1%). This difference between the subgroups within the TDF/FTC/EFV group was seen consistently for most individual neuropsychiatric events recorded.

In the subgroup switched from NVP-based regimens, 41.1% (39/95) of patients in the TDF/FTC/EFV arm experienced a neuropsychiatric event of interest, compared with only 30.6% (30/98) of the patients who switched to the TDF/FTC/RPV arm (Table 4).

**TABLE 4:** Treatment-emergent neuropsychiatric events of interest in the subgroup of patients switching from NVP and the full ITT population.

Adverse event	TDF/FTC/RPV				TDF/FTC/EFV			
	NNRTI at screening: NVP (*n* = 98)		All patients (*n* = 213)		NNRTI at screening: NVP (*n* = 95)		All patients (*n* = 211)	
	*n*	%	*n*	%	*n*	%	*n*	%
Any treatment-emergent neuropsychiatric event of interest	30	30.6	59	27.7	39	41.1	59	28.0
**Nervous system disorders**								
Headache	17	17.3	37	17.4	16	16.8	28	13.3
Dizziness	3	3.1	7	3.3	11	11.6	13	6.2
Somnolence	6	6.1	11	5.2	2	2.1	2	0.9
Hypersomnia	-	-	-	-	1	1.1	1	0.5
Head discomfort	-	-	-	-	-	-	1	0.5
Memory impairment	-	-	-	-	-	-	1	0.5
**Psychiatric disorders**								
Nightmare	4	4.1	4	1.9	6	6.3	9	4.3
Insomnia	5	5.1	10	4.7	2	2.1	4	1.9
Depression	1	1.0	1	0.5	1	1.1	2	0.9
Abnormal dreams	-	-	-	-	1	1.1	1	0.5
Anxiety	-	-	-	-	1	1.1	1	0.5
Libido decreased	-	-	-	-	1	1.1	1	0.5
Libido increased	-	-	-	-	1	1.1	2	0.9
Mood swings	-	-	-	-	1	1.1	1	0.5
Stress	-	-	1	0.5	1	1.1	2	0.9
Restlessness	-	-	1	0.5	-	-	-	-
**Ear and labyrinth disorders**								
Vertigo	6	6.1	11	5.2	12	12.6	16	7.6
**Eye disorders**								
Photophobia	-	-	1	0.5	2	2.1	2	0.9
Vision blurred	1	1.0	1	0.5	2	2.1	2	0.9

EFV, efavirenz; FTC, emtricitabine; NVP, nevirapine; RPV, rilpivirine; TDF, tenofovir disoproxil fumarate.

## Discussion

SALIF examined the effect of switching to TDF/FTC/RPV in patients from LMICs with suppressed viral loads who were on an NNRTI-based first-line ART. This study is important because it provides additional data on the utility of TDF/FTC/RPV as a viable alternative for virologically suppressed patients on first-line NNRTI-based regimen in LMICs and in a study population that is predominantly female because women comprised > 60% of the patients enrolled.

The SALIF data add to the evidence from the SPIRIT study, which examined TDF/FTC/RPV STR as a switch option from a PI-based regimen in mostly Caucasian men in high-income settings,^22^ and the NEAR-RWANDA study, which demonstrated non-inferior efficacy and comparable safety of a TDF/FTC/RPV STR versus NVP-based ART in Rwanda.^25^ Taken together, these data support the use of RPV-based STR regimens in virologically suppressed patients. Given that viral load measurements prior to ART initiation are not routinely conducted in many LMICs, RPV-based STR regimens are an appropriate switch option for patients with suppressed viral loads. These patients have demonstrated sufficient adherence and might benefit from a switch, particularly given concerns around the safety profile of EFV- and NVP-based regimens.

Recent reports from Europe have provided encouraging data on the tolerability of TDF/FTC/RPV in clinical practice, which may be transferable to the LMIC settings.^38,39,40,41,42^ In addition, the introduction of STRs containing tenofovir alafenamide (TAF) 25 mg instead of TDF 300 mg, or an STR of DTG/RPV, could potentially offer increased long-term tolerability at lower dosing and, eventually, lower costs.^43,44,45^ For virologically suppressed patients such as those in the SALIF trial, who are stable on ART and have already demonstrated high adherence, the risk of virologic failure is considered low. Therefore, switching to an STR may further motivate patients to stay on therapy while leading to fewer medication errors and supporting long-term adherence. This is in line with current normative guidance for mature ART programmes, which recommends differentiated models of care for patients who are stable on ART.^46^

Increased rates of rash and neuropsychiatric events have been reported following RPV and EFV treatment;^21,47^ therefore, patients in SALIF were closely monitored for these AEs. In studies in treatment-naive patients, most rash events (3% with RPV vs. 14% with EFV) occurred during the first 48 weeks of treatment,^21^ with few additional patients experiencing rashes during the second year.^47^ SALIF included patients who had already been successfully treated with NNRTIs, and rashes were rarely seen. EFV can cause neuropsychiatric side effects, which often resolve within the first weeks of treatment.^10^ This study could not confirm the improved central nervous system tolerability of RPV compared with EFV seen in treatment-naive patients.^21^ However, the subgroup analysis of patients who switched from NVP to either RPV or EFV showed tolerability differences in favour of RPV.

The strength of this study is that it illustrates the benefit of STRs for patients in resource-limited settings who have tolerability issues with currently available NNRTIs and who are already virologically suppressed. The main limitation of the study was that all patients entering the TDF/FTC/RPV arm changed the previous NNRTI component of their regimen, while > 50% of the patients in the TDF/FTC/EFV arm had previously received an EFV-based regimen. Switching to a new regimen may confer a potential risk for new tolerability or safety issues. Furthermore, the treatment-emergent neuropsychiatric events in the subgroup of patients switching from EFV are discordant with general tolerability data (that show that EFV is associated with higher rates of neuropsychiatric events compared with other NNRTIs, including NVP).^48^ This suggests that our safety findings are subject to some inherent bias of the study design. This negative bias might explain why the ITT analysis showed no differences in tolerability, while other studies in treatment-naive patients have demonstrated a generally more favourable tolerability profile of RPV compared with EFV.^6,7,21,22,24,47^ A control group of non-switchers (or deferred switchers) staying on their original ART might help address such inherent biases in future switch studies. Another limitation of the study is the open-label design, which may influence the reporting of side effects and discontinuation rates. For instance, QT prolongations were reported in three patients on TDF/FTC/RPV and three patients receiving TDF/FTC/EFV; while two of the three patients receiving TDF/FTC/RPV discontinued their regimen, none in the EFV arm discontinued. A caveat to the generalisability of the study results is that trial candidates with CD4 cell counts < 200 cells/mm^3^ were excluded. Also, it should be noted that the definition of viral suppression used in the study was < 400 copies/mL rather than < 50 copies/mL; this was chosen to reflect real-life practice in LMICs and to account for blips, and is within the recommended WHO guidance to use a definition < 1000 copies/mL in LMICs. Finally, our study required participants, as an inclusion criterion, to have access to at least one meal a day, a situation that does not necessarily always pertain in sub-Saharan Africa and other LMIC regions.

## Conclusion

In adults from LMICs with suppressed viral load on first-line NNRTI-based therapy, switching to an STR of TDF/FTC/RPV was non-inferior to an STR of TDF/FTC/EFV in maintaining high rates of viral suppression, with comparable safety at 48 weeks. Our findings support the use of TDF/FTC/RPV as a viable alternative to both EFV- and NVP-based regimens in LMICs, where access to a wider variety of affordable ART options is urgently needed.
